# A Comparative Study on the Uptake and Toxicity of Nickel Added in the Form of Different Salts to Maize Seedlings

**DOI:** 10.3390/ijerph121214972

**Published:** 2015-11-30

**Authors:** Jing Nie, Yuqiang Pan, Jing Shi, Yan Guo, Zengguang Yan, Xiaoli Duan, Meng Xu

**Affiliations:** 1 College of Resources and Environmental Sciences, China Agricultural University, Beijing 100193, China; niejing816@126.com; 2 State Key Laboratory of Environmental Criteria and Risk Assessment, Chinese Research Academy of Environmental Sciences, Beijing 100012, China; duan_jasmine@126.com (X.D.); xumeng@craes.org.cn (M.X.); 3 Key Laboratory of Farming System, Ministry of Agriculture, College of Agronomy and Biotechnology, China Agricultural University, Beijing 100193, China; panyuqiang111@163.com; 4 Department of Safety Engineering, China Institute of Industrial Relations, Beijing 100048, China; shijing8008@163.com

**Keywords:** maize, nickel salts, nickel toxicity, nickel accumulation, soil contamination

## Abstract

In soil ecotoxicological studies, a toxic metal is usually added in the form of either an inorganic or organic salt with relatively high solubility. Nitrate, chloride, acetate, or sulfate are commonly considered as valid options for that aim. However, recent studies have shown that different salts of the same metal at the same cationic concentration may exhibit different toxicities to plants and soil organisms. This information should be considered when selecting data to use for developing toxicological criteria for soil environment. A comparative study was carried out to evaluate the toxicity of five nickel (Ni) salts: NiCl_2_, NiSO_4_, Ni(II)-citrate, Ni(CH_3_COO)_2_, and Ni(II)-EDTA (ethylenediaminetetraacetate), on maize seedlings. The plant metrics used were plant height, shoot and root biomass, leaf soluble sugars and starch, and the Ni contents of the shoots and roots. The results indicated that when Ni was added to the soil, toxicity varied with the selected anionic partner with the following toxicity ranking NiSO_4_ < Ni(CH_3_COO)_2_ < Ni(II)-citrate < NiCl_2_ < Ni(II)-EDTA. Taking the plant-height metric as an example, the effective concentrations for 50% inhibition (EC_50_) were 3148 mg·kg^−1^ for NiSO_4_, 1315 mg·kg^−1^ for NiCl_2_, and 89 mg·kg^−1^ for Ni(II)-EDTA. Compared with the Ni in the other salts, that in Ni(II)-EDTA was taken up the most efficiently by the maize roots and, thus, resulted in the greatest toxic effects on the plants. Nickel generally reduced leaf soluble sugars, which indicated an effect on plant carbohydrate metabolism. The outcome of the study demonstrates that different salts of the same metal have quite different ecotoxicities. Therefore, the anionic counterpart of a potentially toxic metal cation must be taken into account in the development of ecotoxicological criteria for evaluating the soil environment, and a preferred approach of leaching soil to reduce the anionic partner should also be considered.

## 1. Introduction

Nickel (Ni) is used in the production of many modern technologies, including stainless steel, electroplating, and batteries. With the rapid development of these industries, Ni pollution is becoming an increasing problem [1]. Nickel is a group-α transition metal, and the nickel salts of greatest commercial importance include nickel chloride, sulfate, nitrate, carbonate, hydroxide, acetate, and oxide [2]. Nickel generally occurs in the divalent state in the environment, where it can be found in a number of organic and inorganic forms. In soils, nickel may be present as either soluble compounds, such as chlorides and nitrates, or insoluble compounds, such as oxides and sulfides. Soluble Ni compounds tend to exhibit greater mobility than insoluble Ni compounds do, and the concentration of Ni in plants and other soil organisms is generally more closely related to soluble forms of Ni in soil. Therefore, soluble Ni compounds are of great concern in developing soil screening levels [3]. 

Nickel is an essential trace element for plants, but excessive Ni levels in the soil can result in toxicity to plants [4]. The common indicators of Ni phytotoxicity to plants include inhibition of germination, leaf spotting, chlorosis, abnormal flower shape, reduced growth of roots and shoots, deformation of plant parts, poor branching, and decreased yield. Nickel also affect various physiological and biochemical processes in higher plants [4], and high concentrations of Ni affect the iron (Fe) status of the plants [5,6]. Plants can take up Ni through the roots by both passive diffusion and active transport mechanisms. After exposure to high levels of soil Ni, the tissue Ni concentrations of plants are usually highest in the roots, with much less Ni being detected in the stems and leaves [7]. Since an excess of soil Ni decreases Fe uptake by plants, excess soil Ni may also reduce the biosynthesis of these metalloenzymes by causing deficiencies of essential metals [7]. 

In ecotoxicity tests for heavy metal cations, several anion partners are usually employed, including chloride (Cl^−^), nitrate (NO_3_^−^) and sulphate (SO_4_^2−^) [8]. In recent work by Amari *et al.* [4] on Ni accumulation in *Mesembryanthemum* *crystallinum* and *Brassica juncea*, NiCl_2_ was used as the Ni source and was found to inhibit plant growth. Other authors have also used NiCl_2_ as the Ni^2+^ source, including Ma *et al.* [9], who assessed the effects of Ni-mobilizing bacteria during plant growth. Additionally, in 2007, Madhaiyan *et al.* [10] showed that inoculating plants with microorganisms (*Methylobacterium oryzae* and *Burkholderia* sp.) can reduce the toxicity of Ni (added as NiCl_2_) to plants. Additionally, in 2007, Rooney *et al.* [11] used NiCl_2_ to study phytotoxicity in barley and tomato plants and found that root length growth in sixteen European soils was affected by Ni. The effective concentration for achieving 50% inhibition (EC_50_) of root length ranged from 52 to 1929 mg·kg^−1^ and from 17 to 920 mg·kg^−1^ for barley and tomato, respectively. In 2011, Li *et al.* [12] studied Ni toxicity in relation to barley roots elongation, using a total of 17 Chinese soils, again treated with NiCl_2_ as the Ni source. They found that the EC_50_ value for barley roots ranged from 48 to 2519 mg·kg^−1^ among Chinese soils. Other researchers have used NiSO_4_ as the Ni source [13,14,15]. Aydinalp and Marinova [5] studied the effects of Ni^+2^ on alfalfa and found that 20 mg·kg^−1^ of Ni (added as Ni(NO_3_)_2_) significantly reduced seed germination and plant growth. 

Although there have been a number of ecotoxicological studies using a range of Ni source compounds, it remains unclear whether Ni compounds with different associated anions exhibit different toxicities in plants. At this stage, in relation to soil criteria and ecotoxicological testing, the toxicities of nickel chloride, nickel acetate, and nickel sulfate are assumed to be the same. However, if the threshold levels of soil Ni anions are to be established as environmental criteria, a full understanding of the role of the chemical form of a particular Ni pollutant must be achieved. Recently, researchers have noted the influence of anions on heavy metal toxicity. In 2012, Puttaswamy and Liber [16] used the three-brood daphnid *Ceriodaphnia dubia* test to assess the effects of each of the three anions, HCO_3_^−^, Cl^−^, and SO_4_^2−^, on metal toxicity in coke. They showed that inorganic anions have a significant influence on the release of metals from coke, with SO_4_^2−^, especially, increasing the mobilization of metals such as Ni, Fe, Mn and Zn, they also found that HCO_3_^−^, Cl^−^ and SO_4_^2−^ significantly influenced both the mobilization and the toxicity of Ni in oil sands coke. Peredney and Williams [17] showed that the Cl^−^ forms of Ni and Cu were the most toxic, while the NO_3_^−^ forms of Cd and Pb were the most toxic. Hence, it is highly likely that the toxicity of Ni varies with different anions. 

To assess the impact of different anions on Ni toxicity to plants, we carried out a pot experiment using maize and five different Ni compounds, including two inorganic Ni salts (NiCl_2_ and NiSO_4_), and one organic Ni salt (Ni(CH_3_COO)_2_). 

We also used two Ni chelates (Ni(II)-EDTA and Ni(II)-citrate) because EDTA and citric acid are both chelants and are widely used for soil remediation to increase plant uptake by rendering soil metals more soluble. In previous studies, there have been conflicting results regarding the toxicity of Ni(II)-EDTA and Ni(II)-citrate to plants [18,19]. In 2004, Molas and Baran [18] investigated Ni toxicity in barley using four different Ni-source compounds and found that both absorption and phytotoxicity in plants presented the following order: NiSO_4_ > Ni(II)-citrate > Ni(II)-Glu > Ni(II)-EDTA. However, Jean *et al*. in 2008 [19] found that EDTA was most effective in increasing Ni uptake. Therefore, further research on the two Ni chelates is necessary to clarify the influence of EDTA and citric acid on Ni toxicity. 

Through pot experiments, we assessed the toxicity of the five Ni compounds in relation to the growth of maize seedlings, calculating EC10, EC20, and EC50 values of Ni compounds for plant height and biomass. 

The concentration of soluble sugars is an important physiological characteristic of leaves and is often used to study relationships between photosynthesis and yield [20]. Thus, the effects of different Ni compounds on soluble sugars were also evaluated. Soluble sugars are not only major photosynthetic products of higher plants, but are also the main end product of carbohydrate metabolism and temporarily storage. The concentrations of soluble sugars vary with genotype and stress, with sucrose and glucose acting as substrates for cellular respiration and also as osmotic agents [20]. Chen *et al*., in 2014 [21], found that at low concentrations (9–30 mg·L^−1^), when the concentration of chromium (Cr VI) increased, the levels of total proteins and soluble sugars in *Typha angustifolia* increased. Consequently, we used leaf sugar and starch to examine the impact of low and high concentrations Ni (NiCl_2_) and different nickel salts in maize. 

## 2. Materials and Methods 

### 2.1. Materials

Pot experiments were carried out in a soil research greenhouse at the Chinese Research Academy of Environmental Sciences, Beijing, China. Fluvo-aquic soil was collected from the upper soil layers (0–20 cm) from a nearby orchard. The air-dried soil was ground and screened using a 2-mm sieve. The soil had the following characteristics: pH 7.39 and cation exchange capacity 7.20 cmol·kg^−1^; and showed the following particle-size distribution: 73.25% sand, 15.12% silt, and 11.63% clay. The organic matter content was 1.13%. The soil’s baseline concentrations of As, Hg, Cu, Cd, Cr, Zn, Ni, and Pb were 7.3, 0.1, 19.0, 0.3, 56.5, 78.5, 23.1, and 26.0 mg·kg^−1^, respectively [22]. The following analytical-grade chemicals were selected as representatives of soluble Ni salts for the experiments: NiCl_2_·6H_2_O, NiSO_4_·6H_2_O, Ni(CH_3_COO)_2_·4H_2_O, all with purities above 98% (Shanghai, China). Ni(II)-EDTA was prepared by mixing equimolar amounts of Na_2_EDTA and NiCl_2_·6H_2_O. The intense blue color that appeared after mixing indicated a very rapid formation of the complex [18]. Ni(II)-citrate was prepared by mixing equimolar amounts of citric acid and NiCl_2_·6H_2_O [18]. The maize *(Zea mays)* cultivar “ZD 958” was used in the experiment [22]. 

### 2.2. Experimental Design 

#### 2.2.1. Pot Experiments

There were two types of pot experiments. NiCl_2_·6H_2_O, NiSO_4_·6H_2_O and Ni(II)-EDTA were used to generate concentration-response curves in the first pot experiment. A series of NiCl_2_·6H_2_O solutions in deionized water were spiked to the soils at 100 mL·kg^−1^, with the concentrations calculated to result in a logarithmic series of soil Ni contents: 0, 32, 56, 100, 180, 320, 560, and 1000 mg N·kg^−1^ soil dry weight. For NiSO_4_·6H_2_O, 100 mL·kg^−1^ volumes were spiked into the soils to achieve soil dry weight of 0, 180, 320, 560, 1000, 1780, 2500, and 3160 mg Ni·kg^−1^. For Ni(II)-EDTA, 100 mL·kg^−1^ volumes were spiked into the soils to achieve soil dry weights of 0, 100, 180, 320, 180, 320, and 560 mg Ni·kg^−1^. 

The second pot experiment was conducted to assess the uptake and phytotoxicity of five nickel salts in maize. Five Ni compounds (NiCl_2_·6H_2_O, NiSO_4_·6H_2_O, Ni(CH_3_COO)_2_·4H_2_O, Ni(II)-citrate, and Ni(II)-EDTA) were similarly spiked into the soil to give 560 and 1000 mg Ni·kg^−1^ soil dry weight. There were four replicates of each treatment.

After spiking, the soil was thoroughly mixed with a soil blender (NJ-160, Shanghai Shangmai Electronic Instruments, Shanghai, China) and amount of spiked soil equivalent to 2 kg of dry soil was placed in each plastic pot (21 cm upper diameter, 20 cm high). Deionized water was then added to raise the soil moisture content to 20% (w/w) and the pots were left to equilibrate for seven days at room temperature. 

Maize seeds were soaked in deionized water overnight (12 h), and 10 selected seeds were sown 2 cm deep in each pot. The pots were then placed in random order in a greenhouse (23–28 °C, relative humidity 60%, natural lighting), with each pot standing in its own dish [22]. The pots were supplied with nitrogen (N), phosphorous (P), and potassium (K) fertilizer once, immediately following seeding, and were watered daily. The seedlings were thinned to two similar plants per pot, five days after emergence. The plants were harvested 28 days after emergence [22], and the height was measured using a tape measure. 

#### 2.2.2. Maize Growth and Biomass Measurement

The seedlings were harvested and further separated into leaves, stems and roots, and their biomasses (dry weight) were determined. For the determination of biomass, the leaves, stems and roots were dried at 90 °C in a ventilated oven for 30 min, and were then dried to constant weight at 60 °C for 48 h. The plant materials were finally weighed, employing a five-point (0.1 mg) electronic balance (Sartorius, ALC-110.4, Germany).

### 2.3. Measuring Ni in Soil and Plants

One soil sample of 20 g (wet weight) was collected from each pot after the experiment (28 days). The soils were then air-dried, ground, and passed through a 0.15 mm nylon sieve. Subsamples (0.2 g) were taken and placed in 9 mL of HNO_3_:HCl:HF mixture (volume ratio: 5:2:2; 98% HNO_3_; 38% HCl; 47% HF) and digested in a microwave unit (CEM MARS 5, Matthews, USA). The digestion temperature was initially set at 120 °C for 2 min, then raised to 160 °C for 10 min, and finally raised to 180 °C for 20 min [22]. The digestion solution (approximately 8 mL) was transferred to a digestion tube, and 1 mL of HClO_4_ (70%) was added. This mixture was evaporated to a volume of 1 mL at 150 °C on a hot plate, then cooled and transferred to a 50 mL volumetric flask. A sufficient amount of 1% HNO_3_ was added to the solution to obtain a volume of 50 mL, followed by filtering. The concentration of Ni was measured using an Inductively Coupled Plasma Mass Spectrometer (ICP-MS, Agilent 7500c, Santa Clara, CA, USA). Blanks and standard test soil (GBW07410) (National Information Infrastructure for CRMs, China) were measured for quality control. 

The concentrations of Ni in the roots, stems, and leaves were also determined. Briefly, dried samples were cut up with scissors. Samples (0.2 g) for Ni determination were digested for 2 h at 150 °C in 6 mL HNO_3_:H_2_O_2_ (volume ratio 5:1) on a hot plate and then evaporated to a volume 1 mL, cooled and transferred to a 25 mL volumetric flask. The solution was made up to 25 mL with 1% HNO_3_ and subsequently filtered. The concentrations of Ni were measured via ICP-MS. Blanks and certified reference material (tea; GBW10052) (National Information Infrastructure for CRMs, China) were used for quality control. 

### 2.4. Determination of Soluble Sugars, Sucrose, and Starch Concentrations in the Leaves 

For the determination of soluble sugars, sucrose, and starch in the leaves, leaf samples were dried to constant weight, then ground and screened. The powdered material was analyzed using the anthrone colorimetric method to determine soluble sugars and sucrose content [23], and glucose used as a standard to measure starch through colorimetric analysis [24] in a UV spectrophotometer (UV-1800PC, Shanghai, China). 

### 2.5. Determination of Soil pH and EC 

The soil samples were air-dried, ground, and sieved (2 mm pore, nylon sieve). Soil pH was measured using a pH meter (Heriba pH/10N meter F-23), and soil EC (electrical conductivity) was measured using an electrical conductivity meter (Mettler Toledo S700 SevenExcellence^TM^, Switzerland). 

### 2.6. Statistical Analyses

Plant height and biomass were normalized based on comparison with the controls using the following Equation (1):

(1) $$re=\;\frac{re_{t}}{re_{c}}$$

where *re_t_* is the height or biomass (leaf, stem or root) of different treatments, and *re_c_* is the height or biomass (leaf, stem, or root) of the controls (*i.e*., with no added Ni to the soil). 

The effective Ni concentrations causing 10%, 20%, and 50% (*i.e*., EC_10_, EC_20_, and EC_50_) reductions in plant growth (relative height and biomass) were determined using a logistic regression analysis with interpolation to fit the data where there was no significant increase (*p* > 0.05) in the response at low Ni concentrations [12].

(2) $$y\;=\;\frac{y_{o}}{1\;+\;e^{b(x\;+\;a)}}$$

where *y* refers to the relative height and biomass of maize; *x* is the logarithm of the Ni concentration (mg·kg^−1^); *y_o_* and *b* are curve fitting parameters, *y_o_* refers to the maximum response of the fitted curve, *b* is the slope in the log-logistic curve; and *a* is the logarithm of EC_x_ (*EC_x_* is the effective concentration of added Ni causing a 10%, 20%, 50% reduction in height or biomass) [12]. 

Hormesis is the stimulation of a response that can occur at low Ni concentrations and is inhibited at higher concentrations. The EC_10_, EC_20_ and EC_50_ were determined using Equation (3) to account for hormesis [25].

(3) $$y\;=\;c\;+\;\frac{d-c+fx}{1+{\left(\frac{x}{e}\right)}^{b}}$$

where *y* refers to the relative height and biomass of maize; *d* is the relative height and biomass of control (*i.e*., *d* = 1); *c* is the minimum effect; *e* is the concentration at which d − c is reduced by 10%, 20%, or 50% (EC_10_, EC_20_ or EC_50_), *b* is the slope in the log-logistic curve, and *f* is the rate of stimulation [25]. 

A logistic regression analysis was carried out using SYSTAT 11 software. The data were pre-processed using MS Excel; statistical analyses were carried out using SPSS 16.0, and figures were created using the Sigma Plot 12.0 software. 

## 3. Results 

### 3.1. Effects of the Concentrations and Chemical form of Ni on Plant Growth

Regardless of the chemical forms of the Ni salts, maize seedling mortality was only observed at high Ni concentrations, such as 3160 mg·kg^−1^ (NiCl_2_) and 1000 mg·kg^−1^ (Ni(II)-EDTA). However, seedling growth and development were significantly inhibited at high Ni concentrations (1000 mg·kg^−1^ and above). High soil Ni concentrations resulted in dwarfing compared with the control, and the leaves exhibited chlorosis and yellow spotting. 

**Figure 1:**
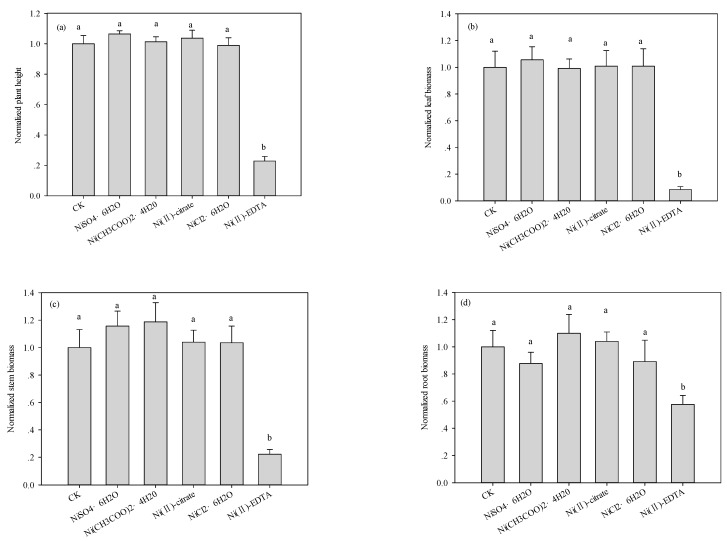
Effects of different Ni compounds added to soil (560 mg·Ni kg^−1^) on the (**a**) plant height; (**b**) leaf biomass; (**c**) stem biomass; and (**d**) root biomass of maize seedlings at 28 days after emergence. The normalized values for the different treatments were calculated in reference to the control (CK), and the error bars represent standard the errors of the means. Values with different letters are significantly different (Duncan multiple range test, *p* = 0.05).

The five Ni compounds had different influences on plant height and biomass at the same soil Ni concentration. At 560 mg·kg^−1^, there was no significant difference between NiSO_4_, Ni(II)-citrate, Ni(CH_3_COO)_2_, and NiCl_2_ in relation to plant height or plant biomass, whereas Ni(II)-EDTA produced a significant decrease (Figure 1). The results of a Duncan multiple comparison show that Ni(II)-EDTA significantly (*p* < 0.05, *n* = 5) decreased plant height and biomass at 560 mg·kg^−1^ compared to the controls and the other Ni treatments. 

**Figure 2:**
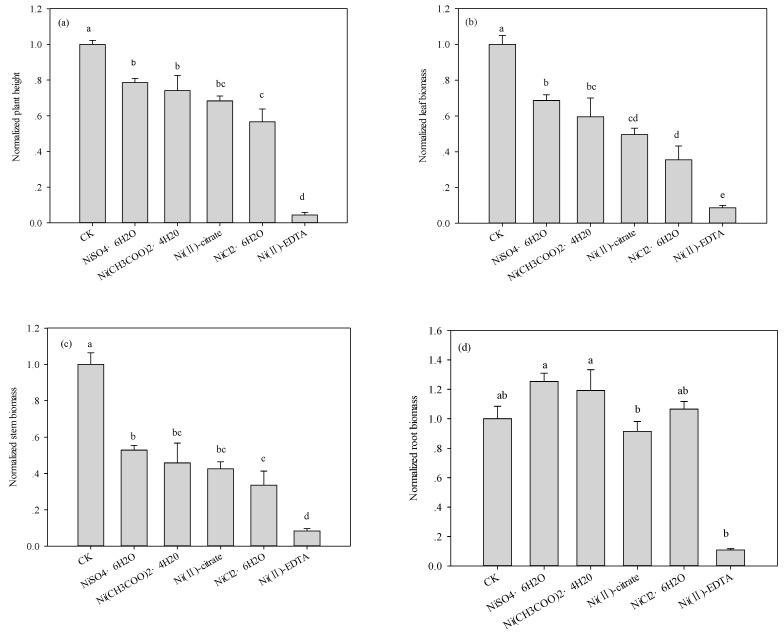
Effects of different Ni compounds added to the soil (1000 mg Ni·kg^−1^) on the (**a**) plant height; (**b**) leaf biomass; (**c**) stem biomass; and (**d**) root biomass of maize seedlings at 28 days after emergence. The normalized values for different treatments were calculated in reference to the control (CK), and the error bars represent the standard errors of the means. Values with different letters are significantly different (Duncan multiple range test, *p* = 0.05).

At high Ni concentration (1000 mg·kg^−1^), there were significant differences in plant height, leaf, and stem biomass between the five Ni compounds treatments (Figure 2). However, the difference in root biomass were not significant. The rank of toxicity in descending order was as follows: Ni(II)-EDTA > NiCl_2_ > Ni(II)-citrate > Ni(CH_3_COO)_2_ > NiSO_4_. This result indicates that the five Ni compounds produced different toxicities at high concentration (≥1000 mg·kg^−1^). Compared with the plant height and biomass of the control, higher Ni concentrations (1000 mg·kg^−1^) significantly (*p* < 0.05) inhibited maize growth (Figure 2). 

**Figure 3:**
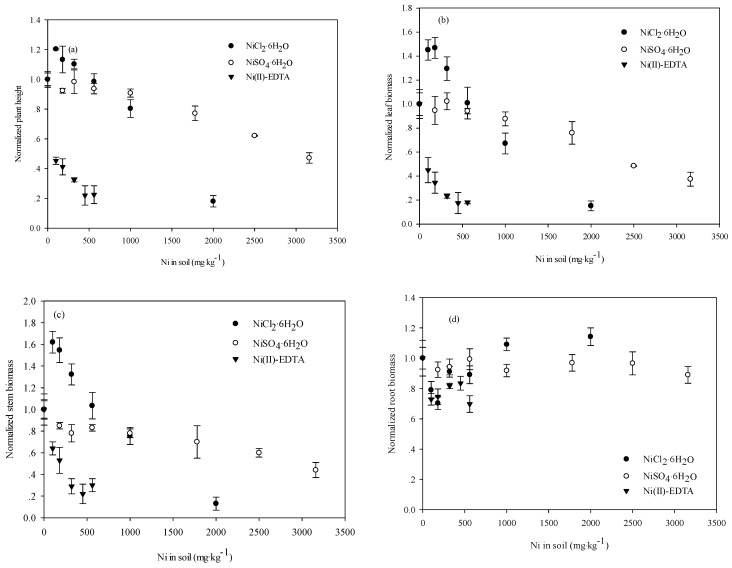
The dose-dependent response of the (**a**) plant height; (**b**) leaf biomass; (**c**) stem biomass; and (**d**) root biomass of maize seedlings to NiCl_2_·6H_2_O, NiSO_4_·6H_2_O, and Ni(II)-EDTA added to the soil. Maize seedlings were sampled at 28 days after emergence. The normalized values for different treatments were calculated in reference to the control, and the error bars represent the standard errors of the means.

Dose-dependent response of plant height and leaf and stem biomass were observed for NiSO_4_, NiCl_2_, and Ni(II)-EDTA added to the soil. Plant height and leaf and stem biomass decreased with increasing soil Ni concentration, whereas root biomass exhibited no significant changes (Figure 3). The Ni EC_50_ values for plant height and stem and leaf biomass varied significantly between NiSO_4_, NiCl_2_, and Ni(II)-EDTA (Table 1). Ni(II)-EDTA presented the lowest Ni EC_50_, whereas NiSO_4_ showed the highest. The Ni EC_10_, EC_20_, and EC_50_ for maize height were 801, 929, and 1315 mg·kg^−1^, respectively, for NiCl_2_; 1303, 1782, and 3148 mg·kg^−1^ for NiSO_4_; and 11, 20 and 89 mg·kg^−1^ for Ni(II)-EDTA (Table 1). Comparatively, soil Ni was the most phytotoxic as Ni(II)-EDTA and the least phytotoxic as NiSO_4_. 

**Table 1:** Effective concentrations of Ni added to the soil as NiCl_2_·6H_2_O, NiSO_4_·6H_2_O, and Ni(II)-EDTA in relation to plant height and the leaf and stem dry weight of maize seedlings.

Test Endpoint	NiCl_2_			NiSO_4_			Ni(II)-EDTA		
	EC10 (mg·kg^−1^ )	EC20 (mg·kg^−1^)	EC50 (mg·kg^−1^)	EC10 (mg·kg^−1^)	EC20 (mg·kg^−1^)	EC50 (mg·kg^−1^)	EC10 (mg·kg^−1^)	EC20 (mg·kg^−1^)	EC50 (mg·kg^−1^)
Plant height	801.7 (477.5–1349.0)	929.0 (626.6–1374.0)	1315.2 (970.5–1778.3)	1303.2 (1000–1698.2)	1782.4 (1503.1–2118.4)	3147.7 (2824.9–3507.5)	10.9 (3.2–36.7)	20.3 (6.6–62.5)	89.1 (45.6–174.2)
Leaf dry weight	465.6 (153.6–1363.8)	751.6 (534.6–1056.8)	962.2 (655.0–1661.6)	1177.6 (847.2–1626.8)	1563.1 (1241.7–1967.9)	2600.2 (2322.7–2917.4)	14.9 (9.9–22.4)	25.5 (17.7–36.9)	83.8 (66.8–105.2)
Stem dry weight	665.3 (335.7–1321.3)	755.1 (396.3–1438.8)	1153.5 (564.9–2349.6)	1000.0 (309.7–3235.9)	1566.8 (743.0–3296.1)	3597.5 (2094.1–6180.2)	33.3 (20.0–103.3)	56.8 (21.9–146.6)	170.2 (96.3–300.6)

Notes: ^a^ Ranges given in parentheses are 95% confidence intervals. ^b^ EC_10_, EC_20_, and EC_50_: the effective concentration at 10%, 20%, and 50% inhibition.

**Table 2:** Ni contents of maize roots, stems, and leaves after 28 days of exposure to different Ni compounds (560 mg Ni·kg^−1^ of soil) in soil.

Parameter	Control	NiSO_4_·6H_2_O	Ni(CH_3_COO)_2_·4H_2_O	Ni( II )-citrate	NiCl_2_·6H_2_O	Ni(II)-EDTA
Ni in soil (mg kg^−1^)	23.1 ± 0.7 ^b^	515.3 ± 10.3 ^a^	524.7 ± 5.5 ^a^	525.3 ± 5.2 ^a^	519.7 ± 4.6 ^a^	530.0 ± 14.4 ^a^
Ni in maize root (mg kg^−1^)	9.8 ± 0.7 ^d^	1548.3 ± 65.8 ^b^	825.7 ± 22.5 ^c^	1314.3 ± 52.1 ^b^	1521.0 ± 29.5 ^b^	2205.0 ± 26.1 ^a^
Ni in maize stem (mg kg^−1^)	0.9 ± 0.1 ^b^	32.4 ± 1.9 ^b^	16.3 ± 0.4 ^b^	25.3 ± 1.1 ^b^	32.4 ± 1.9 ^b^	1703.5 ± 62.5 ^a^
Ni in maize leaf (mg kg^−1^)	1.1 ± 0.2 ^b^	36.9 ± 1.3 ^b^	11.5 ± 0.5 ^b^	23.6 ± 1.5 ^b^	33.0 ± 2.4 ^b^	824.5 ± 36.4 ^a^

Notes: Data in the table are the means ± SE. Different letters (a, b, c and d) in the same row indicate a significant difference (Duncan’s Multiple Range Test, *p* = 0.05).

### 3.2. Accumulation of Ni in Plants

The concentrations of Ni in roots, stems, and leaves of maize plants were determined in relation to treatment with 560 mg·kg^−1^ of Ni added as NiSO_4_, Ni(CH_3_COO)_2_, Ni(II)-citrate, NiCl_2_, and Ni(II)-EDTA (Table 2). The nickel compounds followed a trend in which the highest concentrations of Ni were observed in the roots, with mean concentrations ranging from 826 to 2205 mg·kg^−1^ for the five Ni compounds. The concentrations of Ni in stems and leaves were much lower (<37 mg· kg^−1^) for the treatments with NiSO_4_, Ni(CH_3_COO)_2_, Ni(II)-citrate, and NiCl_2_, whereas that for the treatment using Ni(II)-EDTA, the levels were extremely high (stems 1703 and leaves 824 mg·kg^−1^). The mean Ni concentrations in the three organs decreased in the order: root > stem > leaves. Among the treatments using the various Ni compounds, Ni accumulation was observed in the following descending order: Ni(II)-EDTA > NiSO_4_ ≥ NiCl_2_ > Ni(II)-citrate > Ni(CH_3_COO)_2_.

**Figure 4:**
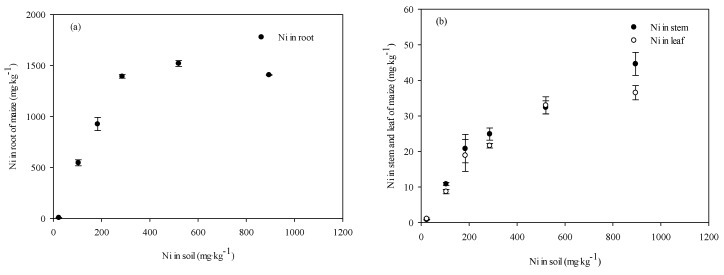
Tissue Ni concentrations (Mean ± SE, n = 5) in the (**a**) roots and (**b**) stems and leaves of maize seedlings after exposure to NiCl_2_ added to the soil. Maize seedlings were sampled 28 days after emergence.

Figure 4 shows the dose-dependent accumulation of NiCl_2_ in the shoots and roots. With increasing soil Ni concentrations, the Ni concentrations in the roots, stems, and leaves also increased. Under the same soil Ni concentration, the roots accumulated higher concentrations of Ni than the leaves and stems; the maximum Ni concentrations in the stems and leaves were less than 50 mg·kg^−1^, but in the roots, the maximum was almost 1500 mg·kg^−1^. The nickel concentrations in leaves and stems were not significantly different (Figure 4). These results indicated that the accumulation of Ni by maize plants occurred mainly in the roots, with only a small amount of Ni being transported to the shoots. 

### 3.3. Soluble Sugars, Sucrose, and Starch in Leaves 

The influence of the four Ni compounds, NiSO_4_, Ni(CH_3_COO)_2_, Ni(II)-citrate, and NiCl_2_ on leaf soluble sugars, sucrose, and starch was determined, and the results were shown in Table 3. In the 560 mg·kg^−1^ Ni treatments with four Ni salts, there were slight reductions of soluble sugars. Leaf starch concentrations in plants exposed to four Ni salts were generally higher than those in the controls, but no significant difference, which was the same with leaf sucrose. 

**Table 3:** Effects of Ni added to the soil (at 560 mg Ni·kg^−1^ of soil) as NiSO_4_·6H_2_O, Ni(CH_3_COO)_2_·4H_2_O, Ni(II)-citrate, and NiCl_2_·6H_2_O on the content of leaf soluble sugars, sucrose and starch.

Parameter	Control	NiSO_4_·6H_2_O	Ni(CH_3_COO)_2_·4H_2_O	Ni(II)-Citrate	NiCl_2_·6H_2_O
Leaf soluble sugar (mg·g^−1^)	75.2 ± 3.4 ^a^	66.0 ± 3.7 ^a^^b^	66.7 ± 4.7 ^a^^b^	62.1 ± 2.6 ^b^	61.4 ± 1.8 ^b^
Leaf sucrose (mg·g^−1^)	9.4 ± 1.3 ^a^	14.1 ± 5.9 ^a^	9.0 ± 2.3 ^a^	6.8 ± 0.9 ^a^	11.1 ± 2.2 ^a^
Leaf starch (mg·g^−1^)	60.6 ± 11.0 ^a^	75.9 ± 8.5 ^a^	75.5 ± 10.4 ^a^	70.1 ± 9.6 ^a^	74.6 ± 4.7 ^a^

Notes: The data are means ± SE. Different letters in the same row indicate significant difference (Duncan’s Multiple Range Test, *p* = 0.05)

**Figure 5:**
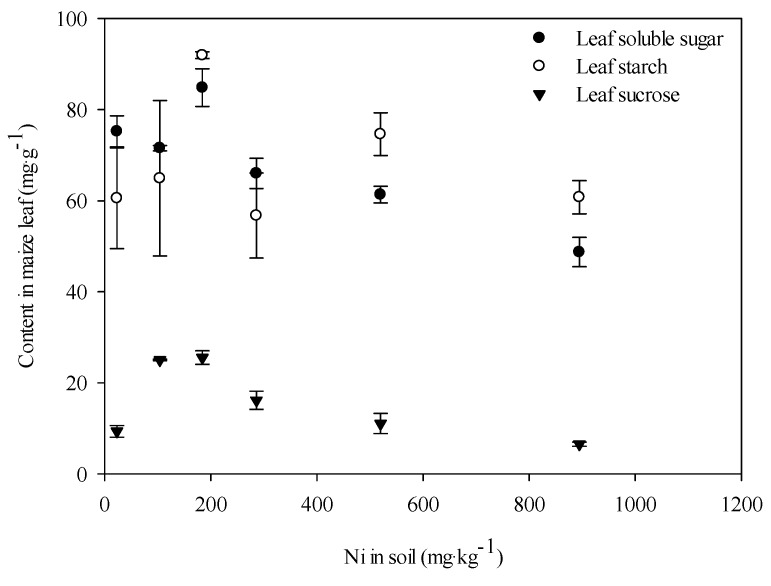
Effects of Ni (added as NiCl_2_) on the leaf soluble sugar, sucrose, and starch contents of maize.

The dose-dependent response of leaf soluble sugars, sucrose, and starch to Ni (added as NiCl_2_) were also determined. The contents of soluble sugar decreased with increasing soil Ni (Figure 5), indicating that Ni inhibited the synthesis of soluble sugar in maize leaves. 

However, the concentrations of sucrose and starch showed no significant changes at different concentrations of Ni in addition to the lower Ni concentrations (180 mg·kg^−1^). At the Ni treatment of 180 mg·kg^−1^, the sucrose and starch data showed significant increases and then appeared to decrease back to the same level as the control. This finding indicates that low Ni concentrations stimulate leaf sucrose and starch, and high concentrations of Ni do not inhibit the synthesis of leaf sucrose and starch. 

### 3.4. Soil pH and EC 

The characteristics of soil including pH and the soil electrical conductivity (soil EC) were measured. In this experiment, there were no significant correlations found between soil pH and toxicity of Ni compounds. The soil pH ranged from 7.27 to 7.64 under treatment with the five Ni compounds (560 mg·kg^−1^) and from 7.56 to 7.20 at the different concentrations of NiCl_2_ (32–3160 mg·kg^−1^ of soil). 

The soil EC values of five nickel compounds treatments (560 mg·kg^−1^) ranged from 0.37 to 1.19 dS/m. the soil EC values of CK, NiSO_4_, Ni(CH_3_COO)_2_, Ni(II)-citrate, NiCl_2_, and Ni(II)-EDTA were 0.56, 0.91, 1.19, 0.68, 1.13, and 0.37 dS/m, respectively. The minimum value was observed for Ni(II)-EDTA and the maximum for Ni(CH_3_COO)_2_. 

## 4. Discussion

Our research indicated that the different Ni compounds added to the soil exhibit different toxicities to the growth of maize seedlings. In our study, there were several reasons for the different toxicities between NiCl_2_, NiSO_4_, and Ni(CH_3_COO)_2_. The first reason was the absorption ability by the plants; and the second one was the ionic strength of cationic metal binding. The additions of metal salts to soils can have an effect on the ionic strength (electrical conductivity) of the soil. In 2011, Li *et al.* [12] reported that high ionic strength can reduce cationic metal binding and make metals more bioavailable; salt stress can cause toxicity in plant, too. Generally, with the increasing of salt content, the soil EC value also increased. 

Ni(CH_3_COO)_2_ has the weakest absorption ability by plant and the highest soil EC value in the three nickel salts (Table 2). Although NiCl_2_ and NiSO_4_ have the similar absorption ability (Table 2), but the soil EC value of NiCl_2_ is higher than NiSO_4_, so the toxicities caused by salt stress of NiCl_2_ is different with NiSO_4_. 

We also confirmed that chelate Ni(II)-EDTA and Ni(II)-citrate had different toxicity, and the absorption and phytotoxicity of Ni(II)-EDTA were far greater than Ni(II)-citrate. Which conflicted with Molas and Baran [18], who studied the uptake and phytotoxicity of Ni compounds, and found the following *different* order: NiSO_4_ > Ni(II)-citrate > Ni(II)-EDTA (75 mg Ni·kg^−1^ of soil), but our conclusion agreed with Jeans *et al.*, in 2008 they also suggested that EDTA is the most effective means of increasing the uptake of Ni than citric acid [19]. The soil EC values of Ni(II)-citrate and Ni(II)-EDTA were 0.68 and 0.37 dS/m, respectively. So the EC value of Ni(II)-citrate is greater than Ni(II)-EDTA. 

After the research on the impact of low and high concentrations Ni (NiCl_2_) and different nickel salts to leaf sugar and starch in maize. We found that at low Ni concentrations (0–180 mg·kg^−1^), the concentrations of leaf soluble sugars, starch and sucrose increased (Figure 5).This result indicated that low Ni levels stimulate leaf sugar and starch. In 2014, Chen *et al.* [21] also reported a similar result that the leaf soluble sugars of *T. augustifolia* increased when the concentration of heavy metal (Cr VI) increased from 9 to 30 mg·L^−1^.

High Ni concentrations significantly decreased the concentrations of soluble sugars in maize leaves (Figure 5). The soluble sugar concentration in the leaves decreased when the Ni (NiCl_2_) concentration was higher than 180 mg·kg^−1^ of soil. This finding agreed with Prodo *et al.* [26], who observed that low concentrations of heavy metal enhanced carbohydrate metabolic functions, whereas high concentrations reduced these functions. 

The phytotoxicity effects of elevated concentrations of soil Ni on maize seedlings include reductions in growth and the appearance of leaf spotting and chlorosis. This agrees with earlier reports on Ni toxicity responses, describing inhibited growth and leaf lesions [13,4,27]. Amari *et al*. in 2014 [4] found that the toxicity symptoms of *B. juncea* plants exposed to soil Ni (in the range 0–100 µM NiCl_2_) included chlorosis of young leaves and necrotic lesions on old leaves. Nickel may have both a direct and/or an indirect effect on photosynthesis, and can also adversely affect the accumulation of macro- and micronutrients in the roots [4]. It appears that Ni inhibits cell elongation growth and photosynthesis, and reduces shoot water content due to a reduction of the transpiration rate [4]. 

Soil environmental thresholds are the basis of most environmental quality standards; therefore, the establishment of scientific and rational soil environmental thresholds requires the availability of appropriate data. This information should include basic soil physical and chemical parameters, ecological toxicology data and the soil background distribution of the pollutant. Toxicology data obtained from laboratory experiments employing artificial pollution will be closer to the data obtained in field experiments if leaching-aging factor corrections are made. The addition of exogenous soluble nickel salts causes an increase in the ionic strength of soil and, thus, enhances the toxicity. In 2011, Li *et al.* [12] found that leaching-aging can reduce the content of soil salts that were previously increased due to the addition of nickel salts, and the greatest difference between leached and unleached soils was observed to be approximately nine-fold. This indicates that removing excess ions from the soil solution following Ni addition is important. In our future work, we will attempt to correct the toxicological data obtained for nickel salts in the laboratory by incorporating a leaching-aging factor to bring these results closer to those recorded using Ni-contaminated soils in the field. 

## 5. Conclusions 

A comparative study was carried out to assess the toxicity of NiCl_2_, NiSO_4_, Ni(CH_3_COO)_2_, Ni(II)-citrate, and Ni(II)-EDTA to maize seedlings in soil. The results indicate that different Ni compounds differ significantly in their toxic impacts on maize seedling growth and in relation to the uptake of Ni. The Ni toxicity of these salts to maize seedlings, increased in the following order: NiSO_4_ < Ni(CH_3_COO)_2_ < Ni(II)-citrate < NiCl_2_ < Ni(II)-EDTA. Thus, the phytotoxicity of a Ni salt depends on its concentration. EDTA was the most effective at increasing the toxicity of Ni to the plant, as well as the uptake and transport of Ni. which could explain why Ni(II)-EDTA is more toxic to maize seedlings than the other salts examined here. Nickel can reduce the soluble sugars contents. A strong implication of these findings is that the anionic partner of Ni must be considered when establishing valid environmental criteria. Further research in this field will focus on the mechanisms through which the various anionic partners of Ni, affect its uptake and plant growth. 
